# Dnmt3b Prefers Germ Line Genes and Centromeric Regions: Lessons from the ICF Syndrome and Cancer and Implications for Diseases

**DOI:** 10.3390/biology3030578

**Published:** 2014-09-05

**Authors:** Emma L. Walton, Claire Francastel, Guillaume Velasco

**Affiliations:** CNRS UMR7216, Epigenetics and Cell Fate, Université Paris Diderot, Bâtiment Lamarck, 4ème étage Case Courrier 7042, 35 rue Hélène Brion, 75205 Paris, France; E-Mails: ewalton86@googlemail.com (E.L.W.); guillaume.velasco@univ-paris-diderot.fr (G.V.)

**Keywords:** DNA methylation, DNMT3B, germ line genes, centromeric repeats, ICF syndrome, cancer

## Abstract

The correct establishment and maintenance of DNA methylation patterns are critical for mammalian development and the control of normal cell growth and differentiation. DNA methylation has profound effects on the mammalian genome, including transcriptional repression, modulation of chromatin structure, X chromosome inactivation, genomic imprinting, and the suppression of the detrimental effects of repetitive and parasitic DNA sequences on genome integrity. Consistent with its essential role in normal cells and predominance at repetitive genomic regions, aberrant changes of DNA methylation patterns are a common feature of diseases with chromosomal and genomic instabilities. In this context, the functions of DNA methyltransferases (DNMTs) can be affected by mutations or alterations of their expression. DNMT3B, which is involved in *de novo* methylation, is of particular interest not only because of its important role in development, but also because of its dysfunction in human diseases. Expression of catalytically inactive isoforms has been associated with cancer risk and germ line hypomorphic mutations with the ICF syndrome (Immunodeficiency Centromeric instability Facial anomalies). In these diseases, global genomic hypomethylation affects repeated sequences around centromeric regions, which make up large blocks of heterochromatin, and is associated with chromosome instability, impaired chromosome segregation and perturbed nuclear architecture. The review will focus on recent data about the function of DNMT3B, and the consequences of its deregulated activity on pathological DNA hypomethylation, including the illicit activation of germ line-specific genes and accumulation of transcripts originating from repeated satellite sequences, which may represent novel physiopathological biomarkers for human diseases. Notably, we focus on cancer and the ICF syndrome, pathological contexts in which hypomethylation has been extensively characterized. We also discuss the potential contribution of these deregulated protein-coding and non-coding transcription programs to the perturbation of cellular phenotypes.

## 1. An Introduction to DNA methylation

DNA methylation was the first described epigenetic mark of eukaryotic genomes shown to affect gene expression, and perturbations to this process were rapidly suspected to cause aberrant transcription in physiopathological situations like aging and cancer [1].

DNA methylation within mammalian genomes is found mainly within the context of CpG dinucleotides. Recent advances in high throughput sequencing have enabled the generation of base pair resolution maps of DNA methylation in an array of different cell types and developmental stages. These maps have unveiled the uneven distribution of DNA methylation, in particular with respect to CpG density [2,3]. They also highlighted the largely context-specific deposition of DNA methylation. The bulk of mammalian genomes is at least partially methylated with the exception of short, CG-rich sequences termed CpG islands, found in around two thirds of promoters [4]. These findings imply that most proximal transcriptional control elements are unmethylated, whereas gene bodies, transposons, and repetitive elements are largely methylated in somatic cells [5]. DNA methylation has been implicated in transcriptional repression [6,7], the control of alternative splicing [8], regulation of transcription from alternative promoters within gene bodies [9], and the inhibition of transposition and mitotic recombination at repetitive sequences [10,11]. It is also involved in X chromosome inactivation in female cells and in the control of allelic-specific expression during genomic imprinting [12,13,14].

DNA methylation is established and maintained by three enzymes with DNA methyltransferase activity, all of which are essential for life [10,15]. Semi-conservative DNA replication provides the opportunity for patterns of existing methylation to be copied onto nascent daughter strands. This is achieved by the activity of the maintenance methylation enzyme DNMT1 [10], which, in complex with its cofactor UHRF1, recognizes and binds to hemi-methylated DNA and accurately copies existing patterns of methylation [16,17]. DNMT1 is ubiquitously expressed during development, in contrast with the *de novo* methyltransferases DNMT3A and DNMT3B, which are highly expressed in embryonic stem cells and are subsequently down-regulated during development in most tissues. These enzymes, in concert with their catalytically inactive cofactor DNMT3L, or alone, depending on the developmental context [18,19], ensure the establishment of methylation patterns from an unmethylated template during early development and thus play an essential role in the correct establishment of methylation patterns [20]. In addition, recent studies have shown a role for *de novo* methyltransferases in the maintenance of methylation patterns at loci such as germ line genes and repetitive elements, suggesting that these enzymes participate in the silencing of these regions in many cellular and developmental contexts [21,22,23].

## 2. DNMT3B: A *de novo* Methyltransferase with Many Isoforms

DNMT3B shares structural organization with DNMT3A. Both proteins possess a variable N-terminal domain, which is followed by a moderately conserved PWWP (Pro-Trp-Trp-Pro) domain [24]. This domain, although it has little or no DNA binding activity, is required for the recruitment of both enzymes to heterochromatin nuclear domains, which occurs through interactions with heterochromatin proteins like HP1 [25]. The PWWP domain is followed by a highly conserved cysteine-rich zinc finger-binding domain called the ADD domain (ATRX-DNMT3-DNMT3L), which shares homology with the plant homeodomain (PHD) and is involved in binding to histone tails [26]. The ADD domain binds to H3 tails unmethylated at lysine 4 (H3K4me3) leading to the preferential methylation of DNA bound to loci with this chromatin state. All DNMTs possess a highly conserved catalytic domain in their C terminus that contains six amino acid motifs, which are involved in distinct steps of the catalytic mechanism including enzymatic catalysis, DNA binding, and S-adenosyl-methionine cofactor binding. Both DNMT3A and DNMT3B can be expressed as alternatively spliced variants in which some of the catalytic motifs or their spacing are disrupted, thus affecting the integrity of the catalytic domain [27,28,29,30,31,32,33].

The *DNMT3B* human gene has 23 exons. The full length DNMT3B1 isoform is strongly expressed during early development and is barely detectable in differentiated cells. Many isoforms have been described for DNMT3B, which result from alternative splicing as mentioned above and/or alternative promoter usage [15]. Notably, the alternative splicing of exons 10 and 11 and exons 21 to 23 within the catalytic domain creates an array of different isoforms, many of which are expressed in differentiated cells, albeit at low levels [20,34,35]. DNMT3B2 lacks Exons 10 and 11 but retains the catalytic domain, whereas DNMT3B3, in addition to Exons 10 and 11, lacks Exons 21 and 22 of the catalytic domain. DNMT3B3 is the most predominant form in somatic cells, although other isoforms are also present, including DNMT3B4 and DNMT3B5, which encode truncated proteins missing parts of the catalytic domain. DNMT3B1 and DNMT3B2 contain all six highly conserved amino acid motifs of the catalytic domain and are enzymatically active. Although DNMT3B3, DNMT3B4, and DNMT3B5 are catalytically inactive, these isoforms may nonetheless interfere with the action of catalytically competent DNMT3B with which they co-exist in the cell, notably in particular pathogenic contexts [29,30]. These inactive isoforms may still retain DNA-binding activity and thus may compete with catalytically active DNMT3B for target binding. Chedin and colleagues demonstrated recently that these variants bind to catalytically competent *de novo* DNMTs and regulate their activity; DNMT3B3 stimulates the basal activity of DNMT3 enzymes, but partially inhibits the stimulatory effect of DNMT3L, whereas DNMT3B4 significantly impairs *de novo* methylation [27].

## 3. Genomic Loci Affected by DNMT3B Loss of Function.

### 3.1. Centromeric and Pericentromeric DNA Repeats

With DNA methylation being non-randomly distributed, and with two *de novo* DNA methyltransferases being implicated in establishment of methylation profiles, one can assume that the enzymes themselves are not randomly recruited to the genome. The identification of genomic regions preferentially methylated by DNMT3B has been largely inferred from studies involving the targeted disruption of the gene in mice and from the analysis of molecular defects observed in human diseases. The first genomic regions shown to be affected by loss of Dnmt3b function were not protein-coding regions, but non-coding repetitive elements found around centromeres [20,36].

In most higher eukaryotes, these regions are assembled on large arrays of tandemly repeated DNA sequences, which can be divided into two distinct functional domains [37]; the centromere *per se*, that serves as the point of assembly for the kinetochore, and flanking heterochromatin domains, thought to protect the centromere and ensure sister chromatid cohesion [38]. In the mouse, pericentromeric domains are made up of A/T rich 234 bp major satellite repeats, and adjacent centromeric regions comprise 120 bp minor satellite repeats [39]. The contribution of these sequences to the genome is substantial, with major satellite repeats representing approximately 3% of the mouse genome and minor satellite repeats representing approximately 0.45% of the genome [40]. In humans, centromeric regions contain 171-bp AT-rich α-satellite motifs. The size and structure of pericentromeric regions varies between chromosomes, and three types of satellite repeats account for about 4%–5% of the human genome. Satellite type I are short AT-rich sequences found at pericentromeric regions of most chromosomes. Type II and type III satellites are made of a 5bp GGAAT repeat unit possibly found on all chromosomes but unevenly distributed over up to several megabases, where they form heterochromatin blocks in pericentromeric regions of certain chromosomes like chromosomes 1, 9, 16 and heterochromatin of chromosome Y [41].

Molecular analysis of mutant mouse embryos lacking Dnmt3b and those lacking Dnmt3a have suggested that Dnmt3b is specifically required for the methylation of centromeric minor satellites whereas Dnmt3a preferentially methylates pericentromeric heterochromatin [20]. The partial loss of function of DNMT3B in patients with the ICF syndrome (see below) is associated with the hypomethylation of pericentromeric satellite type II and III repeats (Sat II, Sat III). The origin of this discrepancy between mouse and human is unclear. Indeed, only general similarities can be drawn from the centromeric regions of the different eukaryotes, such as the abundance of simple or complex repeats, the association of the centromeres with surrounding heterochromatin [42] and the presence of a histone variant, CENP-A, that partially replaces histone H3 in centromeric nucleosomes [43]. In addition, there is a widespread lack of sequence conservation of centromeric and pericentromeric regions throughout the phylogeny, and no conservation of sequence or chromatin organization or even organization in the nuclear space seems to exist between centromeric murine minor satellites and pericentomeric human Sat II and III repeats. Centromeric regions were long considered to be transcriptionally repressed, mainly because they are methylated in somatic cells and because they are surrounded by large blocks of heterochromatin assembled at pericentromeric regions; however, transcripts arising from both centromeric and pericentromeric regions have been characterized in a multitude of organisms and in a variety of cell types and developmental stages (reviewed by [44,45]). These transcripts are integral parts of centromeric and pericentromeric complexes and participate in the assembly and function of centromeres and its surrounding heterochromatin [46,47,48]. Whether they are implicated in the selective recruitment of DNMT3B at murine centromeric and human pericentromeric repeats remains to be tested.

### 3.2. Germ Line Genes

The discovery of additional loci affected by the loss or reduced function of DNMT3B awaited the detailed transcriptional analysis of mouse embryos deficient or partially deficient in Dnmt3b, which revealed the aberrant activation of many genes, of which the most deregulated are normally mainly expressed in the germ line [49,50]. Promoter hypomethylation of these germ line genes strongly correlates with their illicit expression in somatic tissues, consistent with DNA methylation playing the principal role in their transcriptional repression in somatic cells [51,52]. Dnmt3b is present at the promoter regions of several germ line genes in mouse embryonic fibroblasts [50] suggesting that it plays the principal role in the establishment of DNA methylation at these genes during early development. Dnmt3b may even play a role in the maintenance of methylation at a subset of these genes because their methylation status is preserved in the absence of the maintenance enzyme Dnmt1 [23]. Meehan and colleagues have recently demonstrated that the expression of another set of germ line genes involved in genome defense against transposable elements in developing germ cells is also exclusively dependent on DNA methylation mediated by Dnmt3b [53]. Thus DNA methylation is critical for the silencing of a subset of germ line genes and may be the predominant regulatory mechanism at these loci.

### 3.3. Other DNA Repeats

In humans, DNMT3B appears to be the major enzyme involved in the *de novo* methylation of repetitive sequences located in pericentromeric constitutive heterochromatin. The non-satellite repeats NBL2 and D4Z4, which are aberrantly methylated in particular pathological contexts [54,55], are also hypomethylated in DNMT3B deficient cells from ICF patients, suggesting a major role for this enzyme at other repeats with hallmarks of heterochromatin [56]. Likewise, facultative heterochromatin on the inactive X chromosome in cells from female patients and Alu repeats that are widely distributed throughout the human genome are undermethylated in ICF cells [57]. Perhaps more striking is the hypomethylation of sub-telomeric repeats in these patients [58]. However, human telomeres and adjacent subtelomeric regions are packaged as heterochromatin in many organisms and share many epigenetic marks with pericentromeric regions. Human subtelomeric regions are rich in CG sites and these regions are heavily methylated in somatic cells [59].

Thus, in humans, DNMT3B loss of function appears to mainly affect methylation at DNA repeats within or proximal to large heterochromatin domains, consistent with DNA methylation being a hallmark of heterochromatin and DNMT3B playing the principal role in its establishment and maintenance. In the mouse, Dnmt3b cytologically accumulates on constitutive heterochromatin nuclear domains formed by pericentromeric repeats and Dnmt3b loss of function is linked to its delocalization from these domains [60,61]. In this context, it is difficult to understand how this may specifically affect methylation at murine centromeric repeats that form distinct subnuclear domains at the periphery of heterochromatin compartments [37]. Thus, it is possible that human pericentromeric and mouse centromeric repeats share binding sites for factors that are important for the recruitment of Dnmt3b.

## 4. Dnmt3b and Development

### 4.1. Early Development

**Figure 1:**
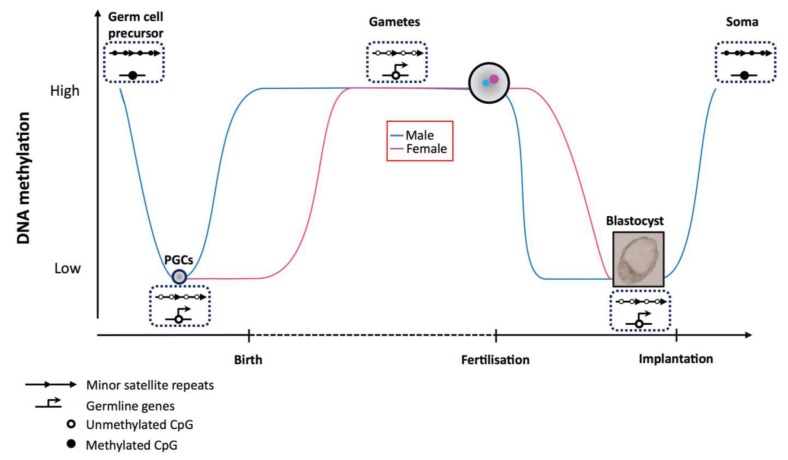
Dynamics of DNA methylation at germ line genes and satellite repeats during the mammalian life cycle. DNA methylation is reprogrammed twice during development; first during the preimplantation blastocyst and again in primordial germ cells. Although germ line genes and minor satellite centromeric repeats are heavily methylated in somatic cells and in germ cell precursors (black circles), they are hypomethylated in primordial germ cells, gametes and in cells of the inner cell mass of the blastocyst (white circles). Global DNA methylation levels are indicated on the y-axis and are shown in blue for the paternal genome and in pink for the maternal genome. DNA methylation at centromeric repeats and germ line genes is established specifically by Dnmt3b during the wave of remethylation that begins around the time of implantation.

DNA methylation is highly dynamic during development and patterns of DNA methylation are reprogrammed genome-wide at two different stages: once in pre-implantation embryos and again at a later time point in developing germ cells (Figure 1).

In the mouse, a wave of methylation erasure occurs in the early embryo [62]. Enzymes of the ten-eleven-translocation (Tet) family have been shown to facilitate DNA replication-independent erasure of CpG methylation by converting 5-methylcytosine (5 mC) to 5-hydroxymethylcytosine (5 hmC) [63,64]. The development of techniques that discriminate 5 mC and 5 hmC has enabled the role of Tet enzymes and that of this potentially new epigenetic mark to be extensively studied in development and disease over the past few years [65,66]. After this step of demethylation, new patterns of DNA methylation are re-established shortly after implantation in the developing epiblast [62]. This *de novo* methylation is required for the differentiation of the epiblast, as shown by the lethality at gastrulation of embryos deficient of both *de novo* methyltransferases Dnmt3a and Dnmt3b [20,67]. However, DNA methylation is not required for establishment and maintenance of naïve pluripotency because embryonic stem cells (ESC) can be established from embryos deficient of the three Dnmts (TKO ESC) [68]. This contrasts with the rapid apoptosis induced by hypomethylation in somatic cells [69].

In murine embryonic pluripotent cells captured in culture, ESC derived from the inner cell mass (ICM) or the late epiblast of post-implantation embryos (EpiSC) do not display quantitative differences in DNA methylation [70]. However, in line with the observed upregulation of the *de novo* methyltransferases Dnmt3a and Dnmt3b, a bias towards methylation of high CpG island promoters in EpiSC was recently described [71]. However, a comparison of EpiSCs and late epiblast cells revealed that the *in vitro* and “*in embryo*” cells show a strikingly different promoter methylation profile [71]. Not surprisingly, these data highlight that the deposition of DNA methylation is greatly affected by culture conditions and that external factors are missing when embryonic stem cells are dissociated and explanted *in vitro*. Along the same lines, when cultured in serum conditions, mouse ESCs fluctuate between a naive ICM-like state and a primed epiblast-like state. However, recent reports have shown that ESCs cultured with inhibitors of the mitogen-activated protein kinase (MAPK) and glycogen synthase kinase 3 pathways (2i), that more faithfully recapitulate the naive state of ICM cells, are significantly more hypomethylated than their serum-cultured counterparts, consistent with low methylation levels found in pre-implantation blastocysts [72,73].

These systems have been widely used to decipher when and where on the genome DNA methylation is established early in development. Major changes occur at the transition from the blastocyst to the post-implantation epiblast, where DNA methylation is primarily targeted to repress the germ line expression program and lineage-affiliated genes such as haematopoietic genes [49]. Dnmt3b catalyses this *de novo* methylation during early embryogenesis, and the absence of DNA methylation leads to ectopic gene activation in the embryo. Methylation at centromeric sequences in ES cells has been more difficult to dissect. In serum-cultured ESCs, many repetitive DNA elements, including centromeric repeats, are highly methylated as they are in somatic cells [74], although, female ESCs are remarkably hypomethylated compared to their male counterparts at both repetitive sequences and unique sequences [75]. In “2i” conditions, maintenance of the naive state appears to be linked to the expression of Prdm14, that acts, at least in part, through down-regulation of Dnmt3b [73,76], recapitulating the low abundance of *de novo* Dnmts described for the ICM *in vivo*. Therefore, repetitive elements, in particular centromeric sequences, are probably hypomethylated in the ICM of pre-implantation embryos.

DNA methylation is reprogrammed a second time in developing germ cells [77]. The majority of the genome is subject to methylation erasure, including imprinted regions and repetitive elements such as centromeric repeats [78,79]. Demethylation enables the activation of the germ line specific program, which as we have seen is largely controlled by DNA methylation. Subsequently, methylation is established with different kinetics in the male and female germ line at time points where the abundance of Dnmt3b is high [80]. However, both germ line genes, which are essential for the establishment of the germ line program, and pericentromeric and centromeric repeats, seem to be protected from this *de novo* methylation in the germ line [81]. Thus, in terms of DNA methylation at these sequences, germ cells closely resemble the pre-implantation blastocyst.

### 4.2. Targeting Dnmt3b during Development

As mentioned above, most CpGs outside CpG islands and regulatory elements are methylated in somatic cells in contrast to the majority of CpG islands that remain unmethylated throughout development. However, CpG methylation is highly dynamic. Although variation may occur stochastically during development and lineage specification, certain CpG islands become selectively methylated, raising the question of how this selectivity is achieved [2,3,82].

Classic studies established that the unmethylated state of promoter CpG islands is strongly influenced by transcription factor binding. For instance, CpG islands can acquire methylation following deletion or mutation of promoter Sp1 binding sites [83,84]. Lienert *et al.* used an approach in which various promoter fragments were targeted to the same genomic insertion site in ESCs to show that DNA methylation is primarily dictated by cis-acting sequences and they identified autonomous methylation determining regions (MDRs) that reside within promoter elements [85]. DNMT enzymes themselves may have a preference for particular DNA sequences flanking target CpG sites [86]. Hervouet and colleagues used transcription factor arrays to systematically identify transcription factors that interact with Dnmt3b [87]. Among these factors, the E2F6 transcriptional repressor is required for the silencing of a subset of germ line genes in somatic cells through the recruitment of Dnmt3b at their promoter region [50] or other repressor complexes in ESCs [88].

Given the lack of sequence conservation of centromeric repeats amongst species, it is highly likely that other mechanisms besides DNA sequence are involved in the targeting of *de novo* methylation to these regions. Indeed, DNMT3B interacts with the centromeric-specific protein CENP-C, which appears to be important for its recruitment to centromeric regions [89]. In addition, there is overwhelming evidence for crosstalk between DNA methylation and chromatin environment. Perhaps the most striking example of this interplay is the inverse correlation between DNA methylation and H3K4 methylation [2,4]. The interaction of the ADD domain with the histone H3 tail is allosterically inhibited by H3K4 methylation [26], thus explaining this association.

The lymphoid-specific helicase LSH (HELLS) is also emerging as a key factor that may be involved in crosstalk between chromatin environment and the establishment of DNA methylation. LSH is a member of the SNF2 family of helicases that are involved in chromatin remodeling [90]. Although mouse embryos lacking Lsh are viable, they show hypomethylation at single copy genes and repeat sequences [91,92]. Genome wide analysis of methylation in cells deficient in Lsh has since documented the severe extent of this hypomethylation [93,94] and has led to the proposition that Lsh may be involved in the recruitment of DNMTs. Specifically, Lsh has been proposed to be involved in the recruitment of Dnmt3b, for example, to the stem cell genes promoter during differentiation of ESCs [95]. This mechanism predicts that *Lsh^−/−^* and *Dnmt3b^−/−^* cells should have similar molecular defects. Indeed, DNA methylation defects in these mutants are strikingly similar [96], notably at particular repeat classes including centromeric repeats. However, hypomethylation at long terminal repeat (LTR) endogenous retroviruses (ERVs) is more severe in *Lsh^−/−^* cells than in *Dnmt3b^−/−^* cells, suggesting that Lsh participates in DNA methylation at particular sequences independently of Dnmt3b [97].

In the developing germ line, small non-coding RNAs are important for the establishment of DNA methylation at transposable elements [98]. Non-coding RNAs that are complementary to the rDNA promoter may also mediate the *de novo* methylation of rRNA genes [99]. Grummt and colleagues showed that a 120 nt stretch of this promoter-associated RNA interacts with the transcription factor TTF-1, forming a DNA:RNA triplex structure that is recognized by DNMT3B. Small non-coding RNA (ncRNA) transcribed from pericentromeric regions are involved in the establishment of heterochromatin, through guiding associated factors, in fission yeast [100]. Although no evidence for such siRNA exists in mammals, long ncRNA transcribed from murine centromeric and pericentromeric regions may represent one of the many pathways that regulate heterochromatin maintenance and assembly in mammals, possibly through directing centromere- and heterochromatin-associated proteins [46,47,48]. Therefore, it is conceivable that such non-coding RNA may also be involved in the targeting of *de novo* methylation to these regions in mammals.

## 5. DNMT3B and Disease

Given the pivotal role that DNA methylation plays during development to regulate gene expression, it comes as no surprise that aberrant patterns of methylation are associated with many human diseases [101]. These may involve isolated events, such as loss of imprinting or changes to the copy number of methylatable trinucleotide repeats, or may entail more global events involving the substantial loss of DNA methylation that is observed in particular immune system disorders including systemic lupus erythematosus and most human cancers [102]. Defects in methylation are associated with a diverse range of clinical outcomes, including intellectual disability, immunodeficiency, autoimmunity, obesity, malignancy, and muscular dystrophy [103]. In cancer, epigenetic changes constitute a highly frequent, arguably invariant hallmark, and cooperate with genetic changes to drive cellular transformation [104]. Perturbations to DNMT enzymes often cause such changes, and the hypomethylation of germ line genes and centromeric regions are common features of cancer cells. Mutations in the DNMT genes are rare in cancer cells, but over-expression of DNMTs is widely described and may partly explain the hypermethylation phenomenon [35]. More recently, splice variants of DNMT3B transcripts have been detected in a wide range of cancer cell lines [28,30,31]. These variants lead to perturbed patterns of methylation which in turn result in the abnormal up or down-regulation of genes. Similarly, in the Immunodeficiency with Centromeric instability and Facial anomalies (ICF) syndrome, germ line mutations in *DNMT3B* lead to perturbed methylation patterns [36]. Both diseases are characterized by the hypomethylation of satellite repeats and germ line genes, in association with chromosomal instability and perturbed nuclear organization, particularly that of heterochromatin nuclear compartments.

### 5.1. DNMT3B Variants in Cancer

One of the first reports to establish a clear connection between DNA methylation and cancer came from Feinberg and Vogelstein in 1983, who found hypomethylation in primary tumor samples [105]. Such reports have led to the idea that the genome of cancer cells is globally hypomethylated. DNA repeats, most notably tandem centromeric α-satellites, juxta-centromeric (centromere-adjacent) Sat II, interspersed Alu repeats, and long interspersed elements (LINE)-1 repeats, largely account for this global DNA hypomethylation [106]. Examples of promoter specific hypomethylation also exist, most notably at the promoters of germ line genes and oncogenes in various cancer cell lines. This hypomethylation is thought to directly lead to their de-repression [51,107]. Germ line genes that are aberrantly expressed in human cancers have also been referred to as “cancer testis” genes because many of them are normally only expressed in the male germ line [108]. Strikingly, this global hypomethylation is concomitant with local hypermethylation, including at the promoter regions of genes involved in cell cycle control or DNA repair, such as p53, Rb, and BRCA1 [104] as well as tumor suppressor genes (TSGs) [109]. Hypermethylation is a major factor in silencing of TSGs and one of the primary inactivating events contributing to tumorigenesis [109].

DNMTs are frequently deregulated in cancers. The general trend appears to be towards the over-expression of these enzymes, which may explain the hypermethylation of TSGs [35]. Nonetheless, somatic loss-of-function mutations in these enzymes have also been described in a wide range of cancer cells. A recent study by Godley and co-workers supports the idea that reduced DNMT3B activity is associated with accelerated tumorigenesis [110]. In this study, inactivation of one allele of *Dnmt3b* in the context of Myc-driven lymphomagenesis resulted in a dramatic increase in the incidence of lymphoma, coincident with alterations in DNA methylation profiles. This suggests that Dnmt3b can act as a haplo-insufficient tumor suppressor gene in the context of myeloid malignancies. Another possible source of altered patterns of DNA methylation in cancer cells is the expression of splice variants. Godley and colleagues documented over 20 DNMT3B transcripts from various cancer cell lines and primary acute leukemia cells that were alternatively spliced at the 5' end of the gene. These variants encode truncated proteins lacking all or part of the C-terminal catalytic domain that may act as dominant-negative isoforms [29]. Indeed, inactive DNMT3B isoforms can alter the enzymatic activity of endogenous full-length DNMT3B *in vitro* [111]. Ectopic expression of the most commonly expressed variant, DNMT3B7, resulted in perturbed expression programs and methylation profiles, including that of the cancer testes gene MAGEA3. Two studies have also correlated the expression of truncated, catalytically inactive variants of DNMT3B, with the hypomethylation of juxta-centromeric Sat II repeats, confirming that DNMT3B splice variants may impair normal enzyme function in a dominant negative fashion, not only at unique gene loci but also at repetitive sequences [30,89]. A direct link between the expression of these isoforms and the hypomethylation of Dnmt3b-target germ line genes has not been examined within the context of cancer cells. Nonetheless, many genes that have been described as targets of Dnmt3b in the mouse [49,50], including *MAEL*, *SYCE1*, and *SYCP1*, are aberrantly expressed in human cancers and are listed as cancer testes genes [112].

### 5.2. Germ Line Mutations in DNMT3B in the ICF Syndrome

Germ line gene mutations in *DNMT3B* are responsible for the autosomal recessive disease named Immunodeficiency with Centromeric instability and Facial anomalies (ICF syndrome) [20,36,113]. ICF is an incredibly rare disease and was initially distinguished based on cytogenetic abnormalities, namely multi-branching of juxta-centromeric regions of chromosomes 1, 9, and 16, detectable in blood cell cultures of patients presenting with primary immunodeficiency and recurrent infections [114,115]. ICF is a clinically heterogeneous disease, although it is almost invariantly characterized by a severe impairment to humoral immunity involving hypo- or agamma-globulinemia. B and T cell numbers and V(D)J recombination are normal [116], suggesting a defect in lymphocyte maturation or activation at late stages. This immunodeficiency leads to recurrent infections that often cause death at a young age. Other clinical features of ICF are present at varying degrees of penetrance and have been extensively reviewed by Hagleitner *et al.* [117]. Facial dysmorphisms are usually mild and typically involve a flat nasal bridge, hypertelorism (widely spaced eyes), epicanthic folds, macroglossia (enlarged tongue), micrognathia (small jaw), and low set ears. Intellectual impairment and neurological defects may also be present, and include slow cognitive and motor development and psychomotor impairment.

The characteristic chromosomal instabilities that are observed in mitogen-stimulated peripheral blood cultures are used for diagnostic purposes. Decondensation of juxtacentromeric heterochromatin of chromosomes 1, and 16, and to a lesser extent chromosome 9, leads to a wide variety of chromosomal anomalies including chromosome breaks and rearrangements, whole-arm deletions, and often multi-branched chromosomes containing three or more arms of chromosome 1 and 16 joined in the vicinity of the centromere [114,115,118,119,120]. Sat II juxtacentromeric repeats on chromosomes 1 and 16 and Sat III juxtacentromeric repeats on chromosome 9 are hypomethylated in cells from ICF patients, thus mimicking an embryonic-like methylation pattern [121]. Treatment of pro-B cells with the DNA demethylating agent 5-azacytidine, but not other non-demethylating genotoxins, induces ICF-characteristic pericentromeric chromosomal anomalies, suggesting that hypomethylation causes these anomalies [122].

ICF is a genetically heterogeneous disease [123,124]. Fifty to 60% of cases are explained by mutations in *DNMT3B*, referred to as ICF1 patients. All affected individuals are homozygotes or compound heterozygotes, mostly for mutations in the C-terminal portion of the protein that contains the catalytic domain [125]. This suggests that ICF is related to impairment in DNA methyltransferase activity and not some other function of DNMT3B. Some residual enzymatic activity is undoubtedly indispensable for survival however, because nonsense mutations are only ever found as compound heterozygotes and complete loss of DNMT3B function is likely embryonic lethal in humans as it is in mice [20]. ICF mutations cause a broad spectrum of biochemical defects in DNMT3B that impair enzymatic activity, including defects in homo-oligomerisation, S-adenosyl methionine (SAM) binding, SAM utilization, and DNA binding [126].

A proportion of ICF patients possess mutations in the *zinc-finger and BTB domain-containing 24* (*ZBTB24*) gene [127], referred to as ICF2 patients. Most mutations create premature stop codons in ZBTB24 suggesting that ICF2 is caused by loss of function of ZBTB24. ZBTB24 is a member of the ZBTB family of transcription factors that are emerging as key regulators of lymphoid development and function (recently reviewed in [128]), however little is known about ZBTB24 itself. Additional cases of ICF remain with no described mutation in either *DNMT3B* or *ZBTB24*, which have been referred to as ICFX [129]. It was initially believed that cases of ICF not related to mutations in *DNMT3B* would be caused by mutations in genes that are functionally related to *DNMT3B*, for example factors that are important for the recruitment of DNMT3B to its target sites. However, molecular and clinical differences between ICF1, ICF2, and ICFX patients are emerging, which is consistent with the involvement of several pathways. In addition to the characteristic Sat II and Sat III sequences, patients without mutations in *DNMT3B* also show hypomethylation of centromeric α-satellite DNA [123]. We have recently reported that the germ line genes *MAEL* and *SYCE1*, which we had identified previously as targets of Dnmt3b in the mouse [50], are hypomethylated and aberrantly expressed in the tissues of ICF1 patients [130]. However, the methylation and expression of these genes was unaffected in uncultured cells from ICF2 and ICFX patients, which suggests that ZBTB24 is not involved in the targeting of DNMT3B to germ line genes and thus is not a simple adaptor of DNMT3B function. Instead, ZBTB24 appears to be involved in targeting DNA methylation to both centromeric and pericentromeric satellite repeats in humans. In the mouse, ectopically expressed Dnmt3b and Zbtb24 are targeted to pericentromeric heterochromatin compartments, independent of pre-existing DNA methylation, whereas this targeting is abolished by mutations found in ICF patients [61,131]. Yet, the impact of *Zbtb24* mutations on the methylation of these repeats has not been determined. We also identified two ICFX patients with molecular characteristics somewhere in between an ICF1 type and an ICFX type [130]. Indeed, these patients showed hypomethylation and expression of germ line genes accompanied by the hypomethylation of α-satellite DNA. The underlying genetic defect(s) in these patients is likely to be highly informative from a mechanistic perspective regarding the establishment and maintenance of DNA methylation. The function of DNMT3B may be impaired in these patients by intronic mutations that cause changes to alternative splicing, or another factor that is required to target DNMT3B to germ line genes. Alternatively, a situation involving dual impairment to DNMT3B and ZBTB24 can also be envisaged. Altogether, these findings highlight the complex etiology of the disease.

Clinical heterogeneity has recently been documented amongst ICF1, ICF2, and ICFX patients, with ICF1 patients displaying a pronounced impairment in humoral immunity and ICF2 patients showing a high incidence of intellectual disability [129]. The identification of molecular differences amongst the various subtypes will be important for the understanding of these phenotypic differences.

## 6. Consequences of Hypomethylation at DNMT3B Targets for Disease

**Figure 2:**
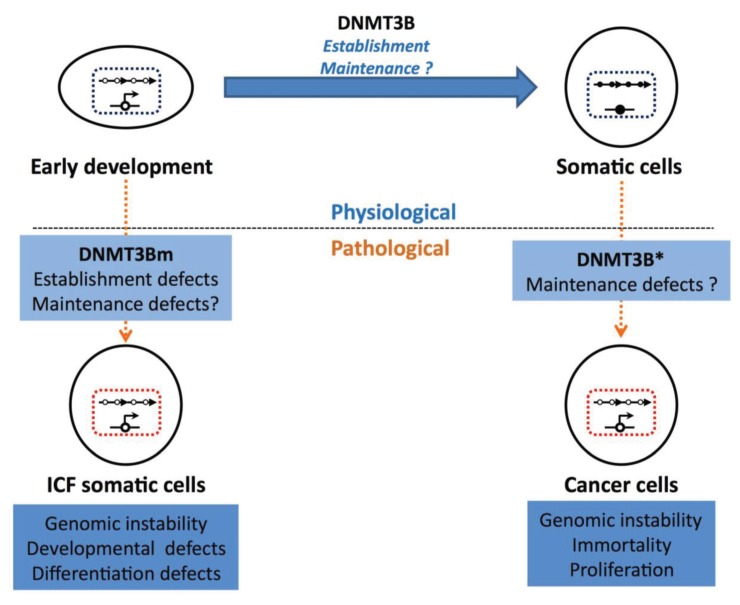
Hypomethylation of germ line genes and satellite repeats in somatic cells as biomarkers for disease. In cancer, the aberrant expression of alternatively spliced *DNMT3B* transcripts (DNMT3B*) lead to the hypomethylation of DNMT3B targets, whereas this is caused by germ line mutations in *DNMT3B* (DNMT3Bm) in the Immunodeficiency Centromeric instability Facial anomalies (ICF) syndrome. As a result, somatic cells from ICF patients or cancer cells show a profile of hypomethylation at germ line genes and centromeric repeats that closely resembles that of ES cells but also gametes. Hypomethylation at these regions is associated with a variety of abnormal nuclear and cellular phenotypes, some of which are common signatures between ICF and cancer cells. Germ line genes and centromeric repeats are represented as in Figure 1. Methylated CpG residues are shown as filled circles and unmethylated CpG residues are shown as open circles.

The hypomethylation of germ line genes and centromeric repeats constitute molecular signatures of the ICF syndrome and cancer (Figure 2) but it is unclear how hypomethylation at these sites create a favorable context for disease. Despite the clear similarities between the molecular signatures of the ICF syndrome and cancer, malignancies have been reported in very few cases in ICF patients [117,132]. In ICF patient cells, in which cell checkpoints appear to be normal [133], it is probable that epigenetic defects alone are not sufficient to drive tumorigenesis. This is consistent with cancer being a “multi-hit” process and the premature death of ICF patients probably does not leave enough time for the subsequent “hits” to occur.

### 6.1. Germ Line Genes and “Meiomitosis”

The hypomethylation and off context expression of germ line genes in somatic cells during carcinogenesis is thought to confer phenotypic traits, including immortality, invasiveness, and immune evasion that are essential for the survival and function of gametes but are hijacked in somatic cells to drive malignancy [134]. In particular, postnatal spermatogenesis is characterized by drastic chromatin rearrangements during meiotic recombination; genome-wide histone hyperacetylation is followed by the replacement of histones with basic proteins called protamines, which leads to the extreme compaction of the genome in mature sperm. Thus, the ectopic activation of this program in somatic cells may create a favorable environment for the alteration of the genome and/or the epigenome [135]. Furthermore, the ectopic expression of germ line (and placental) genes is predictive of poor prognosis irrespective of tumor size, metastasis stage, or histological subtype in human lung cancers [136]. The coexistence of meiotic proteins and mitotic-specific proteins in cancer cells may lead to abnormal chromosome segregation and aneuploidy, a hallmark of human cancer and a driver of genomic instability [137]. Although this has not been formally tested, the ectopic expression of germ line genes drives the growth of malignant brain tumors in *Drosophila* *melanogaster* [138]. In ICF syndrome, the finding that particular germ line genes are also aberrantly expressed in ICF1 patients suggests that they may participate in chromosomal instability in this syndrome. However, the finding that they are not expressed in ICF2 patients [130], raises the possibility that this illicit activation may not only contribute to the pathogenesis of ICF but also to the diversification of ICF phenotypes. This link, however, as well as the potential mechanism underlying it, remains to be established.

### 6.2. Transcription of Satellite Repeats and Chromosomal Instability

During early development in the mouse, satellite sequences are transcribed in the testis and brain [139], in undifferentiated ESCs [140], and in pre-implantation embryos [48]. However, in most adult tissues pericentromeric and centromeric regions are not transcribed, or at low levels. In this context, the transcription of these regions appears to be spatially and temporally regulated throughout cell cycle [47,141], and transcripts that arise from them participate in the establishment of pericentromeric heterochromatin domains and centromere-associated complexes [47,48].

How perturbations to the methylation status of these regions directly affect cellular phenotypes is not known. In addition to the alteration of gene expression programs, the hypomethylation of satellite repeats is believed to contribute to chromosomal instability that is typical of cancer cells and ICF patient cells. An emerging possibility is that hypomethylation leads to the aberrant transcription of underlying repeat sequences, as illustrated by treatment of cultured cells with the DNA demethylating agent 5’azacytidine that promotes the accumulation of pericentromeric transcripts in human cells [142] or centromeric transcripts in mouse cells [46]. In support of this hypothesis, the aberrant accumulation of pericentromeric transcripts has been detected in cancer cell lines [142] and in primary human epithelial cancers [143], as well as in lymphoblastoid cells from an ICF patient [144], consistent with B-lymphocytes being particularly affected in these patients. Likewise, in senescent embryonic lung fibroblasts and epithelial carcinoma cells, hypomethylation and decondensation of the 1q12 locus is associated with a high abundance of pericentromeric transcripts [145]. Although these examples do not give any clue as to the causal link between high levels of satellite transcripts and chromosomal instability, the forced accumulation of satellite transcripts leads to mitotic defects such as multiple spindle attachments, loss of sister chromatid cohesion and aneuploidy [46,146,147]. Yet, the absence of strong constitutive transcription of satellite repeats in most human or murine ICF cells suggests that hypomethylation *per se* is not sufficient to promote their transcription, at least in cultured cells. Overall, there is a clear correlation between DNA hypomethylation of satellite repeats and their strong transcription in certain cellular contexts, although the relationship between methylation and transcription at these regions appears complex. However, this raises the interesting possibility that tissue-specific or developmental stage-specific factors are required, and may explain why all tissues of the ICF patients or mice are not similarly affected.

It is also possible that DNA methylation at centromeric repeats plays additional roles besides transcriptional regulation. The number of centromeric repeats in mouse *Dnmt3a^−/−^Dnmt3b^−/−^* ES cells is lower than in wild-type ES cells, and this is associated with a higher rate of centromere mitotic recombination in *Dnmt3a^−/−^Dnmt3b^−/−^* ES cells [148]. Thus, DNA methylation may be an important mechanism to suppress illicit centromeric mitotic recombination.

The hypomethylation of telomeric and sub-telomeric repeats in ICF patient cells is also associated with abnormally short telomeres in both the telomerase-positive and -negative cells, suggesting that DNA methylation may protect cells from telomere shortening [58]. Furthermore, ICF cells express abnormally high levels of telomeric repeat containing RNA (TERRA), suggesting that methylation is important for keeping transcription of these regions at low levels and that elevated levels of TERRA cause telomere shortening by unknown mechanisms [58].

### 6.3. Hypomethylation of Satellite Repeats and Perturbed Nuclear Organization

**Figure 3:**
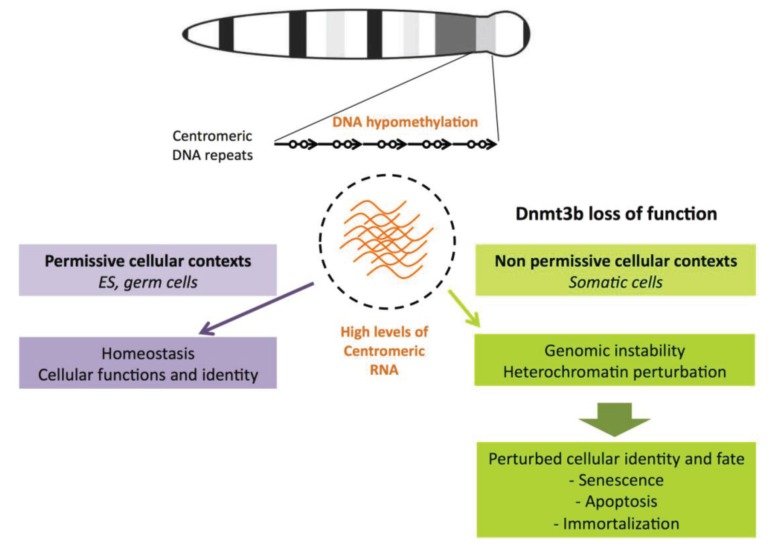
The effect of high levels of satellite transcripts is context dependent. We represent here the example of murine chromosomes and murine cells for which the forced expression of centromeric transcripts leads to phenotypes that are reminiscent of perturbations described for cancer and ICF cells. DNA hypomethylation of centromeric DNA repeats is not sufficient to drive the transcription of these repeats but may define a molecular context that promotes activated transcription in particular cellular contexts. Transcription of these repeats is low in somatic cells whereas high levels have been described in embryonic stem cells (ESCs) and germ cells, although it is not clear whether this is required for their identity. In physiopathological contexts where the function of Dnmt3b is altered and centromeric repeats are hypomethylated, high levels of centromeric transcripts accumulate only in particular somatic tissues. Here, they may trigger molecular defects such as genomic instability and perturb nuclear heterochromatin domains implicated in the repression of gene expression, thus leading to perturbed cellular phenotypes. As a consequence, the abnormal accumulation of satellite transcripts may act as a master switch in diseases characterized by alterations to methylation status, like cancer and ICF syndrome.

Repetitive sequences play an essential role in the three-dimensional organization of the nucleus through the formation of repressive compartments [149,150]. Thus, perturbations to heterochromatin regions, known to have long-range influences on gene regulation through position effects, in cancer or ICF cells, probably have profound effects on gene expression. As discussed above, the transcriptional activation of heterochromatin regions in this context is likely to play an important role in these perturbations (Figure 3).

Alterations to juxtacentromeric heterochromatin resulting from DNA hypomethylation leads to perturbed nuclear heterochromatin and mis-localization of associated proteins [151]. These perturbations may affect gene expression in *trans* and contribute to the developmental defects of ICF patients and to the phenotypic characteristics of cancer cells. The nuclear volume occupied by the juxtacentromeric compartments of chromosomes 1 and 16, along with their positioning relative to the nuclear periphery, differs between cells from ICF patients and those from healthy individuals [152]. The authors of this study confirmed perturbed expression of four genes located on chromosome 1 that were previously identified as deregulated in ICF patient cells [153]. Three of these genes had CpG islands in their predicted regulatory regions but showed no perturbation to promoter methylation. Instead, upregulated genes were more often positioned away from juxtacentromeric heterochromatin of chromosome 1 in ICF patient cells than in control cells. Therefore, by affecting long-range gene-heterochromatin associations, the altered subnuclear organization of the hypomethylated satellite sequences interferes with heterochromatin mediated gene silencing and contributes to some of the changes in gene expression observed in ICF cells.

## 7. Conclusions and Perspectives

The establishment of correct patterns of DNA methylation is essential for normal development, and the loss of or deviation from these patterns is a critical event in many human diseases. Thus, the identification of the targets of *de novo* DNMTs is crucial for our understanding of normal and pathological development, and animal models have been indispensable for the elucidation of methylation patterning.

No clear mechanistic link between genomic hypomethylation and chromosomal instability has been yet established, and cells from ICF patients and ICF mouse models constitute powerful tools to study the complex relationship between DNA hypomethylation and pathological phenotypes, although there are some limitations regarding the sequence conservation between human and mouse centromeric/pericentromeric satellite repeats. They also provide a unique opportunity to study the targets and function of DNMT3B in a human or murine context free from experimental manipulation. Indeed, these systems may be used to examine the role of DNA methylation at satellite repeats, the complex relationship between hypomethylation and transcription of these regions, and the relevance for perturbed molecular and nuclear phenotypes. Many questions still remain about the identity of factors that are important for the establishment of DNA methylation at germ line genes and centromeric repeats and the study of the ICF syndrome is likely to provide some clues to this conundrum. Indeed, human genetic studies of the ICF syndrome have identified a new player in DNA methylation at DNA repeats in centromeric regions, ZBTB24, and functional studies will soon dissect the role of this factor in the establishment or maintenance of methylation. The identification of more factors from the study of ICFX patients seems probable and will contribute to our understanding of how DNA methylation is established and maintained at centromeric and pericentromeric DNA repeats. Finally, further studies of the significance of the off-context expression of DNMT3B targets, in particular centromeric RNA and germ line genes, will help to elucidate the specific contribution of these factors to pathological phenotypes and may even firmly establish them as viable therapeutic targets or powerful biomarkers.
